# Intestinal Enterobacteriaceae that Protect Nematodes from the Effects of Benzimidazoles

**DOI:** 10.4172/2155-9597.1000294

**Published:** 2016-10-31

**Authors:** John H Whittaker, Alan P Robertson, Michael J Kimber, Tim A Day, Steve A Carlson

**Affiliations:** Department of Biomedical Sciences, Iowa State University College of Veterinary Medicine, Ames, IA, USA

**Keywords:** Enterobacteriaceae, Nematode, Anthelmintic, Benzimidazoles

## Abstract

The objective of this study was to investigate an interaction between nematodes and gut Enterobacteriaceae that use benzimidazoles as a carbon source. By addressing this objective, we identified an anthelmintic resistance-like mechanism for gastrointestinal nematodes. We isolated 30 gut bacteria (family Enterobacteriaceae) that subsist on and putatively catabolize benzimidazole-class anthelmintics. *C. elegans* was protected from the effects of benzimidazoles when co-incubated with these Enterobacteriaceae that also protect adult ascarids from the effects of albendazole. This bacterial phenotype represents a novel mechanism by which gastrointestinal nematodes are potentially spared from the effects of benzimidazoles, without any apparent fitness cost to the parasite.

## Introduction

Approximately 2 billion people are infected with gastrointestinal (GI) nematodes worldwide [1]. Benzimidazoles are one of the preferred drug classes for treating these parasites but, unfortunately, resistance is emerging [2]. Resistance is conferred, at least in some instances, by mutations in the nematode genes encoding for tubulin [3], a cytoskeletal protein targeted for inhibition by benzimidazoles [4]. These mutations alter the benzimidazole-binding site on tubulin without hampering the function of the cytoskeletal protein [3].

Resistance to anti-infectives is typically based on: altering the target of the anti-infective (as observed with tubulin alterations and benzimidazole resistance); creation or utilization of a new or alternative pathway that circumvents the target of the anti-infective; efflux of the anti-infective out of the pathogen; or, chemical alteration of the anti-infective drug. The resistance repertoire of nematodes is somewhat limited when compared to bacteria since prokaryotes are capable exhibiting all of these four resistance mechanisms whereas the resistance of nematodes does not usually include alteration of the drug.

Empirically observed resistance to benzimidazoles has not been completely associated with tubulin gene mutations [2]. In a previous study we identified bacteria capable of catabolizing and inactivating antibiotics [5]. In the current study, we examined this possibility for bacteria that subsist on benzimidazoles. Furthermore, we investigated an interaction between nematodes and commensal intestinal bacteria whereby the bacteria may use benzimidazoles as a carbon source and the nematode would thereby be protected from the benzimidazoles.

## Materials and Methods

The bacteria used in this study included isolates obtained from various fecal sources including toilet water, canine feces, bovine feces, feline feces, ovine feces, caprine feces, environmental water, and soil. Samples were first inoculated in 20 mL of Lennox L broth and then grown statically overnight at 37°C. An aliquot (100 µL) of the growth was then plated on XLD agar to partially select for bacteria of the Enterobacteriaceae family, which are frequent commensals of the gastrointestinal tract of eukaryotes [6,7].

In our previous studies we assessed the ability of *Salmonella*, a member of the Enterobacteriaceae family, to subsist on and putatively catabolize antibacterial drugs. To do so, we used single-carbon-source media (SCSM) to assess the ability of the isolates to subsist on a given antibacterial drug [8]. In the current study we used the same approach to identify Enterobacteriaceae that subsist on albendazole (Sigma Aldrich), a model benzimidazole. Specifically, enteric bacteria (approximately 10^8^ CFUs) from each XLD agar plate were collected *en masse* in PBS and transferred onto SCSM-albendazole agar containing 1 µg/mL of the albendazole as per previous studies [5,8]. Bacteria that grew on the SCSM-albendazole (about 5% of samples yielded growth within 24 hrs) were transferred to SCSM-mebendazole, SCSM-fenbendazole, and SCSM-thiabendazole (1 µg/mL of each drug). Bacteria that grew on SCSM-albendazole were also transferred to XLD agar and subjected to a PCR and DNA sequencing procedure that exploits a hypervariable region of 16s rRNA, in order to determine the genus and species of a candidate Enterobacteriaceae [9].

To determine if the subsistence on benzimidazoles extrapolated to an inactivation of the drug, adult *C. elegans* was co-incubated with one of the four drugs and an Enterobacteriaceae (toilet water *E. coli*) capable of subsisting on benzimidazoles. A range of 50–100 adult *C. elegans* strain N2 were placed on NGM agar (100 mm plates) containing 0–200 ng/mL (which includes the estimated range of intestinal drug concentration) of one of four benzimidazoles. Up to 10^9^ CFUs/mL (which includes the estimated range of intestinal Enterobacteriaceae concentration) of a benzimidazole-catabolizing *E. coli* was co-incubated with the *C. elegans*. For all *C. elegans* assays, the total bacterial concentration was maintained at 10^9^ CFUs/mL by the addition of the standard food source of *E. coli* strain OP50 [10]. *C. elegans* were maintained at 25°C in the dark and then were microscopically monitored for movement (or lack thereof) at three days after the addition of bacteria [11]. Controls included worms incubated with non-catabolic isostrains (ATCC), worms not exposed to a benzimidazole, and worms incubated with a catabolic strain plus 200 ng/mL of albendazole in the presence of ambient light that leads to the degradation of albendazole [12]. Percent worm viability (i.e., percent of worms exhibiting motility during a two minute visualization period) was then noted for each *E. coli*-benzimidazole combination.

Statistical differences were examined using an ANOVA with Tukey’s test for multiple comparisons (GraphPad Prism 6.0). p<0.05 was considered to be significant.

## Results

As shown in Table 1, we isolated 30 different Enterobacteriaceae capable of subsisting on albendazole. These isolates represent the following bacteria: *E. coli*, n=10; *Enterobacter aerogenes*, n=6; *Klebsiella pneumonia*, n=5; *Citrobacter koseri*, n=4; *Providencia vermicola*, n=2; *Enterobacter cloacae*, n=2; and, *Klebsiella oxytoca*, n=1. All 30 isolates were capable of subsisting on each of the four benzimidazoles examined.

**Figure 1:**
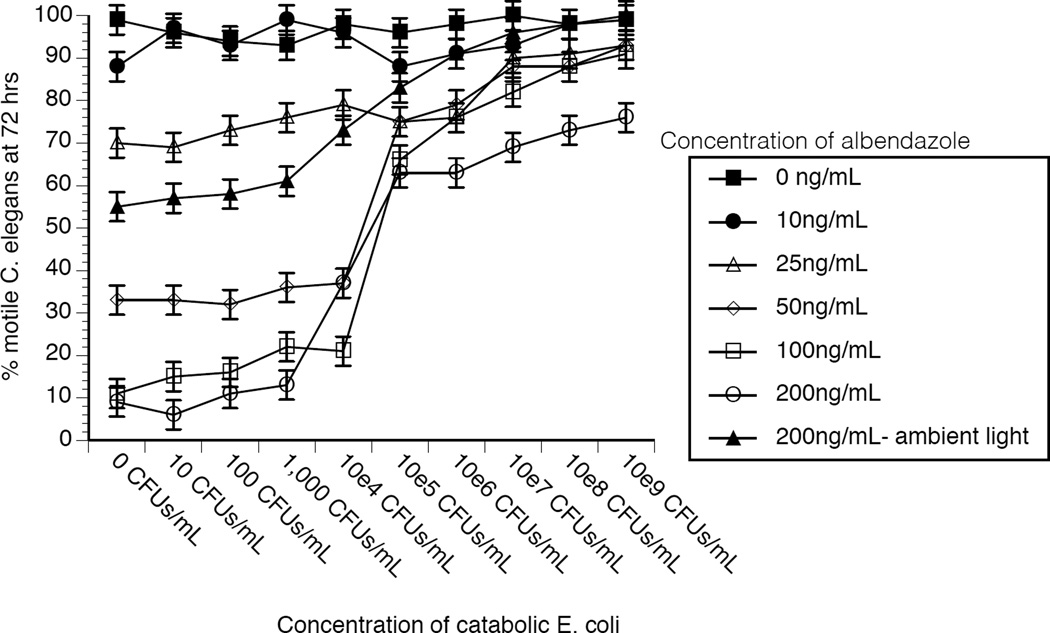
Diminished albendazole effectiveness in *C. elegans* co-existing with a benzimidazole-catabolizing *E. coli*. A range of 50–100 adult *C. elegans* (average of 73 per assay) were co-incubated with various concentrations (0–10^9^ CFUs/mL) of a benzimidazole-catabolizing *E. coli* (toilet water *E. coli* isolate) (Table 1) and 0–200 ng/mL of albendazole. Total bacteria concentration totalled 10^9^ CFUs/mL by the inclusion of food strain *E. coli* strain OP50 (10). Worm activity was then monitored microscopically after 3 days, and % motile worms were calculated. Data represent the mean + sem for four independent experiments. The following concentrations of bacteria significantly (p<0.05) altered % motile worms, when compared to worms exposed to no albendazole at the same concentration of bacteria, at the parenthetically designated concentrations of albendazole: 10^4^ CFUs/mL (200 ng/mL- ambient light); 10^5^ and 10^6^ CFUs/mL (50 ng/mL, 100 ng/mL, 200 ng/mL, and 200 ng/mL- ambient light); 10^7^–10^9^ CFUs/mL (25 ng/mL, 50 ng/mL, 100 ng/mL, 200 ng/mL, and 200 ng/mL- ambient light).

To determine if the subsistence extrapolate to protection for a nematode, adult *C. elegans* were co-incubated with various concentrations of a benzimidazole-catabolizing *E. coli* and albendazole. As shown in Figure 1, the benzimidazole-catabolizing *E. coli* protected *C. elegans* from the effects of multiple concentrations of albendazole, and that the protection afforded by the *E. coli* was dependent upon the density of catabolic bacteria co-incubated with *C. elegans*. The following concentrations of bacteria significantly (p<0.05) altered % motile worms, when compared to worms exposed to no albendazole at the same concentration of bacteria, at the parenthetically designated concentrations of albendazole: 10^4^ CFUs/mL (200 ng/mL- ambient light); 10^5^ and 10^6^ CFUs/mL (50 ng/mL, 100 ng/mL, 200 ng/mL, and 200 ng/mL- ambient light); 10^7^–10^9^ CFUs/mL (25 ng/mL, 50 ng/mL, 100 ng/mL, 200 ng/mL, and 200 ng/mL- ambient light). It is of note that maintaining the worms in ambient light, which degrades albendazole [12], provided protection from albendazole and this effect was amplified in the presence of benzimidazole-catabolizing *E. coli*.

**Figure 2:**
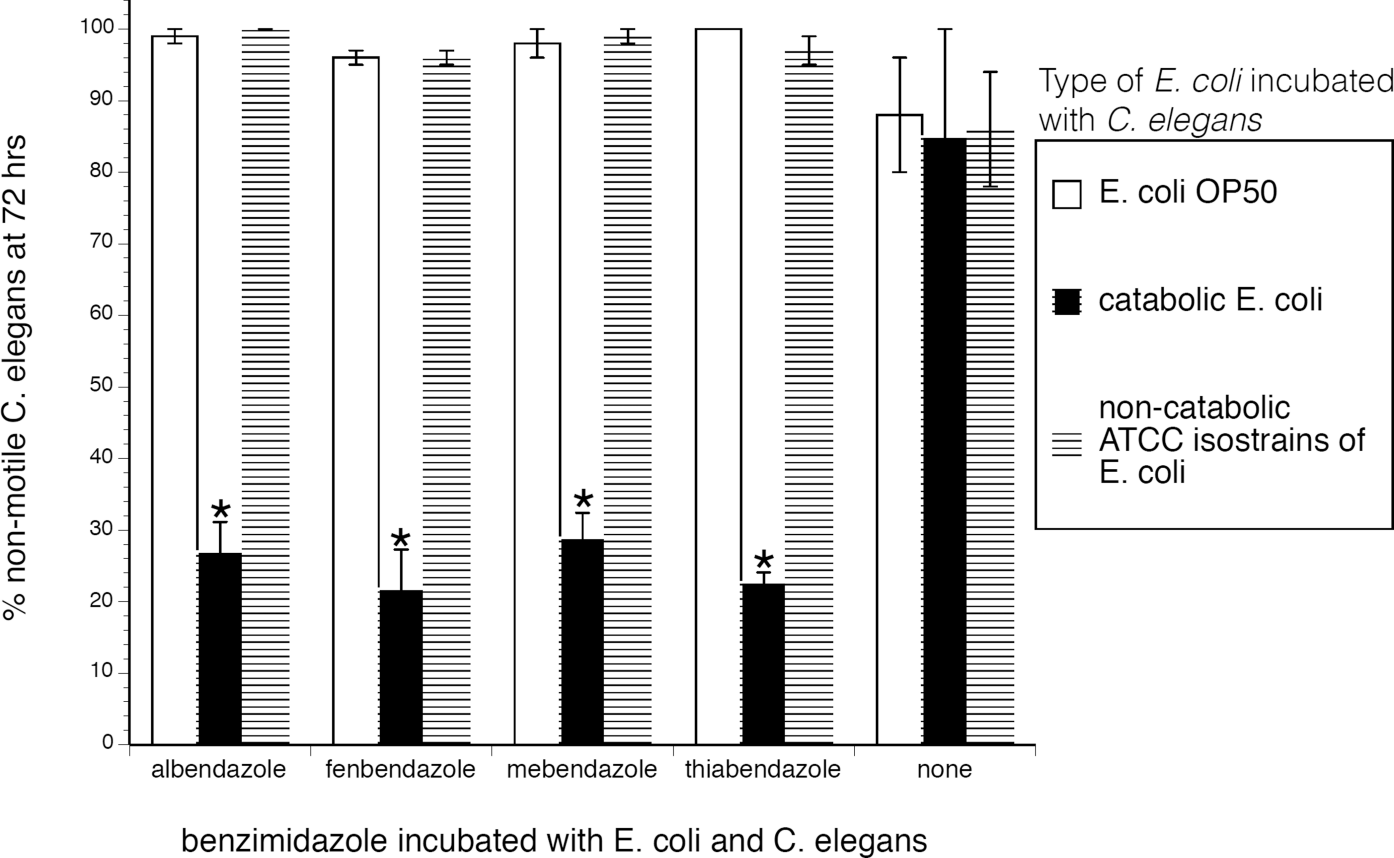
Diminished benzimidazole effectiveness in *C. elegans* co-incubated with benzimidazole-catabolizing *E. coli*. A range of 50–100 adult *C. elegans* were co-incubated with 10^7^ CFUs/mL (lowest tested concentration of bacteria that consistently altered benzimidazole efficacy at most drug concentrations tested as shown in Figure 1) of four different benzimidazole-catabolizing *E. coli* (or *E. coli* OP50) and 100 ng/mL (lowest drug concentration leading to the maximal catabolic response) of a benzimidazole. Worm activity was then monitored microscopically after 3 days, and % of non-motile worms was calculated. Data represent the mean + sem for three independent experiments. *^p^<0.05 vs. non-catabolic bacteria and *E. coli* OP50. For these studies we randomly chose the following *E. coli* isolate/benzimidazole combinations: Nigerian water isolate/albendazole; bovine fecal isolate/fenbendazole; ovine fecal isolate/mebendazole; and, a caprine fecal isolate/thiabendazole (Table 1).

To determine if the observed effect was valid for other benzimidazoles, adult *C. elegans* were co-incubated with four different benzimidazole-catabolizing *E. coli* (or *E. coli* OP50) and a benzimidazole. Worm activity was then monitored microscopically after 3 days, and % of non-motile worms was calculated. As shown in Figure 2, the protective effect extended to three other benzimidazoles: fenbendazole, mebendazole, and thiabendazole. It is of note that the *E. coli* are possibly catabolizing the benzimidazoles on NGM agar even though this media has ample carbon sources for supporting bacterial growth [10].

**Figure 3:**
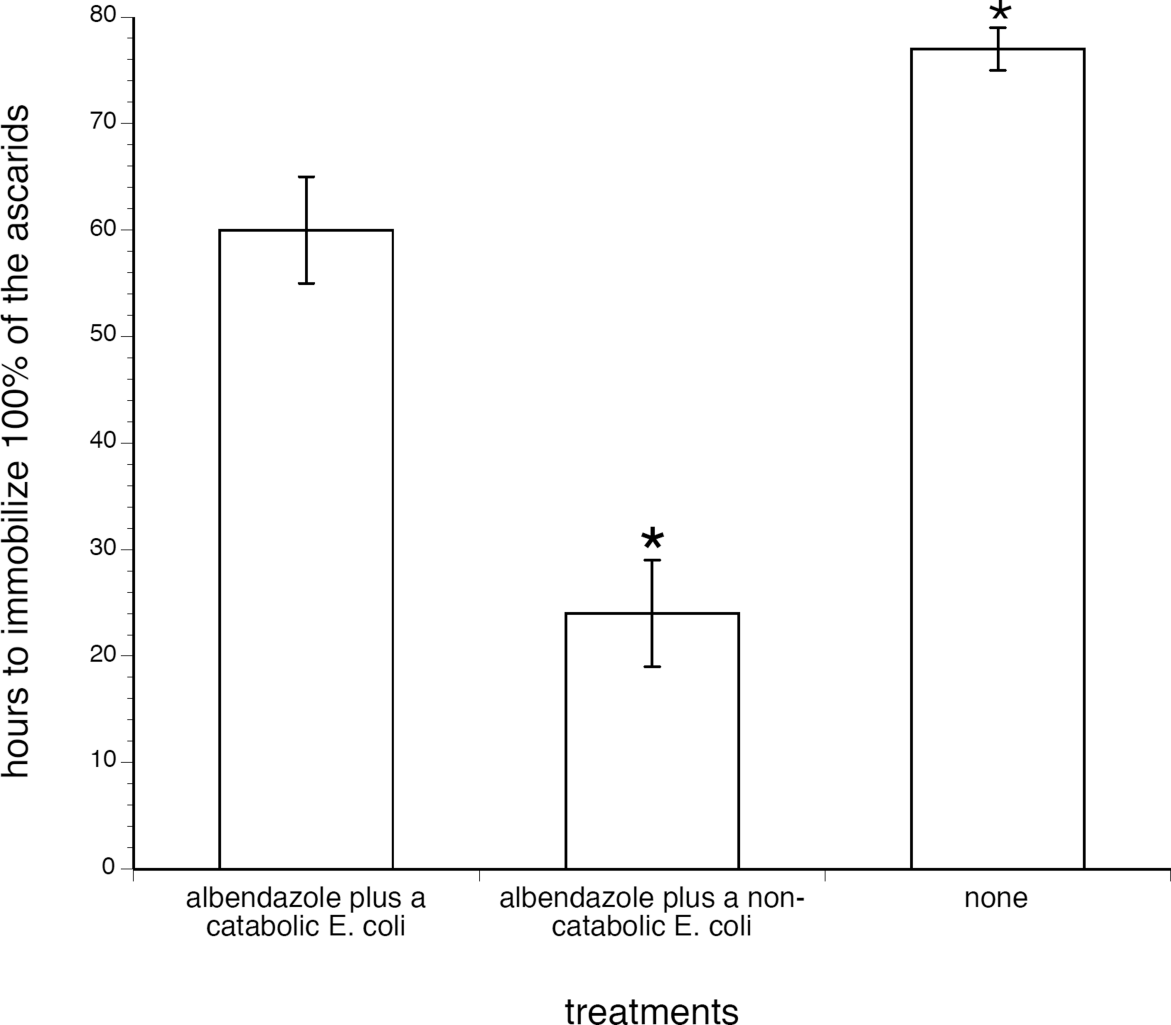
Diminished benzimidazole effectiveness in ascarids co-incubated with a benzimidazole-catabolizing *E. coli*. Adult ascarids were obtained from swine at a commercial abattoir (as previously described by (16)), maintained in RPMI/FBS (Difco; which supports ascarids (17) and the Enterobacteriaceae (18)), and monitored at least every 12 hrs for activity following the addition of albendazole plus a benzimidazolecatabolizing *E. coli*. Specifically, four to five adult worms were incubated with 10^7^ CFUs/mL (i.e., the lowest tested bacterial concentration that consistently altered the efficacy of albendazole as per Figure 1) of one of the benzimidazole-catabolizing *E. coli* (toilet water isolate). Media contained 65ng/mL of albendazole, i.e., the approximated albendazole EC_50_ in the absence of catabolic bacteria as per Figure 1. A control group entailed worms, 65 ng/mL albendazole, and 10^7^ CFUs/mL of non-catabolic bacteria (*E. coli* OP50). Media (including bacteria and drugs) were replenished daily and worm activity was monitored every 12 hr. Time points were recorded in which 100% of the worms were immobile. Values derived are means + sem from assays performed on five different occasions, with fresh batches of worms used each time. *p<0.05 vs. catabolic bacteria (*E. coli* toilet water isolate).

To determine if the observed effects extended to adult gastrointestinal nematodes, adult ascarids were obtained from swine and monitored at least every 12 hrs for activity following the addition of albendazole plus a benzimidazole-catabolizing *E. coli*. Figure 3 reveals that the catabolic bacteria were able to protect swine-derived intestinal ascarids from albendazole. For these studies, ascarids were obtained from swine and incubated with 65 ng/mL of albendazole and a 10^7^ CFUs/mL of catabolic bacterium in liquid media. The catabolic bacteria more than doubled the time required for albendazole to immobilize, as determined by visualize inspection for complete lack of movement over a two minute period, 100% of a population of adult nematodes obtained from the gastrointestinal tract of a mammal.

## Discussion

Herein we report a phenomenon in which gut Enterobacteriaceae are capable of subsisting on benzimidazoles, an effect that provides a passive benzimidazole resistance-like mechanism to free-living and gastrointestinal nematodes living in proximity to the bacteria. This study does not uncover the mechanisms underlying the phenomenon, but it does bring forth the possibility of an emerging problem with benzimidazole failures. That is, this study brings forth the possibility of a new mechanism for treatment failures in gastrointestinal nematodes. The bacteria isolated were obtained from various fecal sources, including the developing world where gastrointestinal nematodes are more prevalent [13]. Our studies suggest that benzimidazoleinsensitive nematodes could appear in intestinal tracts in which the parasite co-exists with commensal Enterobacteriaceae exhibiting the catabolic phenotype. A number of studies have established that Enterobacteriaceae interact with gastrointestinal nematodes, either by living in proximity to the nematode [14] or by physically interacting with the nematode [15]. Additionally, we could find no studies identifying an antagonism between Enterobacteriaceae and gastrointestinal nematodes. Therefore, this relationship does not appear to compromise the fitness of the nematode. However, the initial studies presented herein do not identify a dramatic benefit for the bacteria and thus the effect will disappear when the bacteria are no longer near the nematode.

In summary, this is the first report of a phenomenon in which commensal gut bacteria catabolize benzimidazoles and thus potentially protect local nematodes from the effects of the drugs. Fortunately, this phenomenon has an intervention point that can be pharmacologically targeted. The catabolism-conferring enzymes, once identified, could be pharmacologically inhibited without instigating an adaptive response by the bacteria. That is, the catabolism-conferring enzymes do not appear to be vital for the bacteria and thus inhibiting these enzymes will not induce any selection pressure upon the catabolic bacteria.

## Figures and Tables

**Table 1:** Identities and sources of bacteria capable of subsisting on benzimidazoles.

Genus and species	Sources of the isolates
*E. coli* (n=10 isolates)	Nigerian environmental water; Bovine feces; Canine feces (n=2); Ovine feces; Caprine feces; Toilet water (Iowa State University); Environmental soil (n=2); Feline feces
*Enterobacter aerogenes* (n=6 isolates)	Equine feces; Feline feces; Bovine feces (n=2); Environmental water
*Klebsiella pneumoniae* (n=5 isolates)	Canine feces (n=3); Bovine feces; Raccoon feces
*Citrobacter koseri* (n=4 isolates)	Canine feces (n=3); Environmental soil
*Enterobacter cloacae* (n=2 isolates)	Feline feces; Canine feces
*Providencia vermicola* (n=2 isolates)	Canine feces; Environmental water
*Klebsiella oxytoca* (n=1 isolate)	Equine feces

## References

[1] WHO (2013). Soil-transmitted helminth infections.

[2] Vercruysse J, Behnke JM, Albonico M, Ame SM, Angebault C (2011). Assessment of the anthelmintic efficacy of albendazole in school children in seven countries where soil-transmitted helminths are endemic. PLoS Negl Trop Dis.

[3] Von Samson-Himmelstjerna G, Blackhall WJ, McCarthy JS, Skuce PJ (2007). Single nucleotide polymorphism (SNP) markers for benzimidazole resistance in veterinary nematodes. Parasitology.

[4] Barrowman MM, Marriner SE, Bogan JA (1984). The binding and subsequent inhibition of tubulin polymerization in Ascaris suum (in vitro) by benzimidazole anthelmintics. Biochem Pharmacol.

[5] Barnhill AE, Weeks KE, Xiong N, Day TA, Carlson SA (2010). Identification of multiresistant Salmonella isolates capable of subsisting on antibiotics. Appl Environ Microbiol.

[6] Petersen L, Tisa L (2013). Friend or foe? A review of the mechanisms that drive Serratia towards diverse lifestyles. Can J Microbiol.

[7] Leimbach A, Hacker J, Dobrindt U (2013). E. coli as an all-rounder: the thin line between commensalism and pathogenicity. Curr Top Microbiol Immunol.

[8] Dantas G, Sommer MOA, Oluwasegun RD, Church GM (2008). Bacteria subsisting on antibiotics. Science.

[9] Weisburg W, Barns S, Pelletier D, Lane D (1991). 16S ribosomal DNA amplification for phylogenetic study. J Bacteriol.

[10] Brenner S (1974). The genetics of Caenorhabditis elegans. Genetics.

[11] Navarrete-Vázquez G, Yépez L, Hernández-Campos A, Tapia A, Hernández-Luis F (2003). Synthesis and antiparasitic activity of albendazole and mebendazole analogues. Bioorg Med Chem.

[12] Ragno G, Risoli A, Ioele G, De Luca M (2006). Photo- and thermal-stability studies on benzimidazole anthelmintics by HPLC and GC-MS. Chem Pharm Bull.

[13] Pacifico P (2001). Nematodes: worms of the world. MLO Med Lab Obs.

[14] Chadfield M, Permin A, Nansen P, Bisgaard M (2001). Investigation of the parasitic nematode Ascaridia galli (Shrank 1788) as a potential vector for Salmonella enterica dissemination in poultry. Parasitol Res.

[15] Hayes K, Bancroft A, Goldrick M, Portsmouth C, Roberts I (2010). Exploitation of the intestinal microflora by the parasitic nematode Trichuris muris. Science.

[16] Robertson AP, Clark CL, Burns TA, Thompson DP, Geary TG (2002). Paraherquamide and 2-deoxy-paraherquamide distinguish cholinergic receptor subtypes in Ascaris muscle. J Pharmacol Exp Ther.

[17] Urban JJ, Douvres FW, Xu S (1984). Culture requirements of Ascaris suum larvae using a stationary multi-well system: increased survival, development and growth with cholesterol. Vet Parasitol.

[18] Nietfeld J, Yeary T, Basaraba R, Schauenstein K (1999). Norepinephrine stimulates in vitro growth but does not increase pathogenicity of Salmonella choleraesuis in an in vivo model. Exp Med Biol.

